# Preliminary validity evidence for a platform-specific assessment tool for robotic setup and docking

**DOI:** 10.1007/s11701-026-03589-x

**Published:** 2026-07-20

**Authors:** T. Shakir, G. Lingam, M. Boal, G. Ryan, W. Ghamrawi, M. Chand, N. Francis

**Affiliations:** 1 University College London, London, UK; 2 St Mark’s Hospital and Academic Institute, London, UK; 3 The Griffin Institute, Harrow, UK

**Keywords:** Robotic surgery, Docking, Setup, Validity evidence, Assessment, Accreditation

## Abstract

There is currently no widely available, structured assessment tool with reported validity evidence for robotic system setup and docking. A Delphi consensus-derived checklist for the da Vinci Xi platform has been developed previously. The present study aimed to gather preliminary validity evidence for this checklist using Messick’s unified framework and Kane’s validation inferences. Fifty-one participants without prior robotic experience completed a structured four-day basic robotic skills course. Docking and setup performance was assessed at the start (day 1) and end (day 4) of the course by an independent, ALSGBI-certified robotic trainer using the 69 point checklist. Five dry-lab tasks were assessed at both time points using the Global Evaluative Assessment of Robotic Skills (GEARS) score. Internal structure evidence was obtained from a pre-study calibration exercise and a post-hoc inter-rater reliability analysis in which three independent, blinded raters scored 12 randomly selected procedure videos. Consequences were assessed through Wilcoxon signed-rank comparison of pre- and post-training scores. Relationship to other variables was examined exploratorily comparing checklist and GEARS scores with Spearman correlation coefficient. Inter-rater reliability across 12 videos and three raters demonstrated ICC = 0.91 (95% CI 0.75–0.97) for total scores. Docking scores increased from a median of 43.0 (IQR 34.0–47.5; 62.3%) to 58.0 (IQR 53.0–61.0; 84.1%), a relative increase of approximately 35% (*p* < 0.001; *r* = 0.87). A weak but significant positive correlation between docking and composite GEARS scores was observed at the second time point (Spearman ρ = 0.31, 95% CI 0.04–0.54; *p* = 0.027), with the interrupted suture task showing the strongest individual association (ρ = 0.38, 95% CI 0.12–0.59; *p* = 0.006). This study provides preliminary validity evidence supporting the use of a Delphi-derived checklist for the structured assessment of robotic setup and docking on the da Vinci Xi platform. Further evidence, particularly from multi-centre studies, more raters, and comparison across experience levels, is required before the tool can be recommended for accreditation purposes.

**Supplementary Information:**

The online version contains supplementary material available at 10.1007/s11701-026-03589-x.

## Introduction

Robotic surgery has expanded considerably over the past two decades, with over 3 million robotic procedures performed globally in 2025 [1]. This growth places increasing demands on the training of the entire surgical team, including the bedside assistant, who can be predominantly responsible for patient cart setup, docking, instrument exchange, and emergency undocking; tasks that are technically complex and can influence operative safety [2].

Despite the ubiquity of docking and setup within operating rooms, training in robotic system setup has lacked standardisation. Electronic learning modules, industry-led and surgical societal courses predominate, with variable focus on docking and no uniform competency framework [3]. The absence of structured, platform-specific assessment tools makes it difficult to compare standards across institutions, to deliver formative feedback in a consistent way, or to credential trainee surgeons or surgical care practitioners increasingly involved in robotic bedside assisting [4].

To address this, an international Delphi consensus generated statements describing best practice in robotic system setup and docking on the da Vinci Xi platform. This was performed by international expert participants from 13 countries and included sections on system knowledge, port placement principles, driving in and docking, instruments and changes, and undocking and driving out. These statements were subsequently rationalised into a usable online assessment tool, which requires evaluation.

Contemporary validity theory does not regard an assessment instrument as inherently “valid”; rather, validity is a property of the interpretation of scores in a defined context, supported by accumulated evidence [5, 6]. Messick’s unified framework, adapted by Cook and Hatala [7] for educational assessment, recognises five sources of evidence: content (the relationship between the assessment and the construct), response process (the integrity of the scoring procedure), internal structure (reliability and item behaviour), relations to other variables (convergence and discrimination with related measures), and consequences (the downstream effects of using the tool). A complementary framework described by Kane organises the validity argument around four inferences—scoring, generalisation, extrapolation, and implications [8]. This is well suited to articulating how evidence supports the intended use of a checklist based assessment. Validation is therefore an ongoing, programmatic activity in which evidence is accumulated incrementally across studies.

This prior work established content evidence for the checklist. The present study aimed to gather preliminary validity evidence across the remaining sources of Messick’s framework, with the corresponding Kane inferences, providing a complementary structure for the validity argument, to support the interpretation of scores generated by this checklist in a training context.

## Methods

### Study design and setting

A prospective observational study was conducted at The Griffin Institute, Harrow, UK, a Royal College of Surgeons of England (RCSEng) accredited training centre. Participants attended a structured four-day basic robotic skills course delivered under the auspices of a UK surgical society [9]. Participation in the study was voluntary, and all data were anonymised prior to analysis.

### Training course

**Fig. 1:**
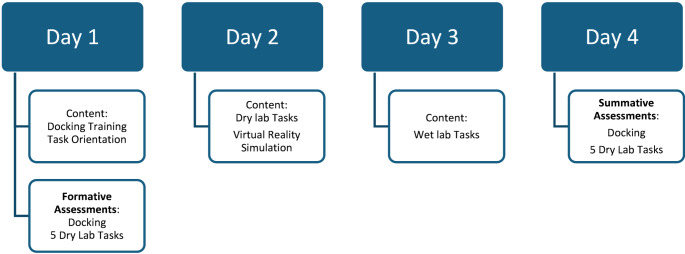
Timetable for societal basic robotic skills course

The four-day course (Fig. 1) is a structured robotic curriculum for novices designed to develop competence progressively across the principal domains of robotic practice. Day 1 comprised a faculty-led training session on robotic system setup and docking using the da Vinci Xi platform, followed by demonstration and supervised practice on five dry-lab tasks and virtual reality (VR) simulator exercises. Day 2 consisted of further dry-lab and VR simulator practice. Day 3 involved wet-lab work, including an avian dissection task and a porcine small-bowel anastomosis. Day 4 comprised additional dry-lab task practice followed by summative assessments.

Two structured docking and setup assessments using the study checklist were undertaken: a formative at the end of day 1 (following the faculty-led training session), and a summative on day 4. Training sessions were delivered in groups of four to six, with group size determined by resource availability.

### Participants

All participants were members of the surgical team with no prior formal training in robotic setup or docking. No exclusion criteria were applied with respect to grade, specialty, or seniority, reflecting the heterogeneous population for whom the checklist is intended. Demographic data collected included age, gender, hand dominance, grade (resident, consultant/attending), years of surgical practice, and number of laparoscopic procedures performed.

### Docking checklist

**Fig. 2:**
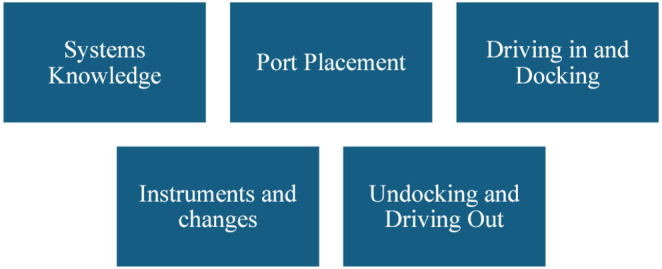
Components of Docking Checklist

The 69-item assessment checklist used in this study was developed previously. An international Delphi consensus [10] was undertaken by a multidisciplinary steering committee (two robotic surgeons, including lead faculty of an ALSGBI societal robotic course; two robotic surgical care practitioners, each with over 1000 cases; and a lead industry representative), which drafted 88 initial statements across five procedural phases. Three rounds of voting were completed by over 50 internationally recognised societal robotic subcommittee members and experienced robotic mentors and assistants almost two-thirds had been involved in over 500 robotic cases. The consensus statements were rationalised into a 69-item observable-behaviour checklist organised across the five Delphi phases [11]—systems knowledge, port placement, driving in and docking, instruments and changes, and undocking and driving out (Fig. 2). Each consensus statement mapped exactly to a checklist item, and was grouped within the aforementioned phases (table in Supplementary material 3). Additional explanatory context to be provided to the candidate was added and was subsequently implemented as an online tool with real-time scoring (Fig. 3, Supplementary material 1).

Each item is scored as present (1) or absent (0), giving a maximum score of 69. Binary scoring was employed for discrete, observable steps to reduce rater subjectivity, consistent with task-based procedural checklists used elsewhere in surgical training [12], with the granularity of the checklist further limiting variability. While individual items are weighted equally, each section contributes a differing amount to the total score. More complex phases, such as port placement and driving in and docking, comprise a greater number of steps and therefore contribute proportionally more. This provides an implicit weighting structure derived from the consensus. Items reflecting basic system knowledge (for example, correctly identifying components) are accessible items appropriate for an entry-level assessment, allowing the checklist to discriminate across a range of performance.

### Online form consisting of 69 point docking checklist with live scoring

**Fig. 3:**
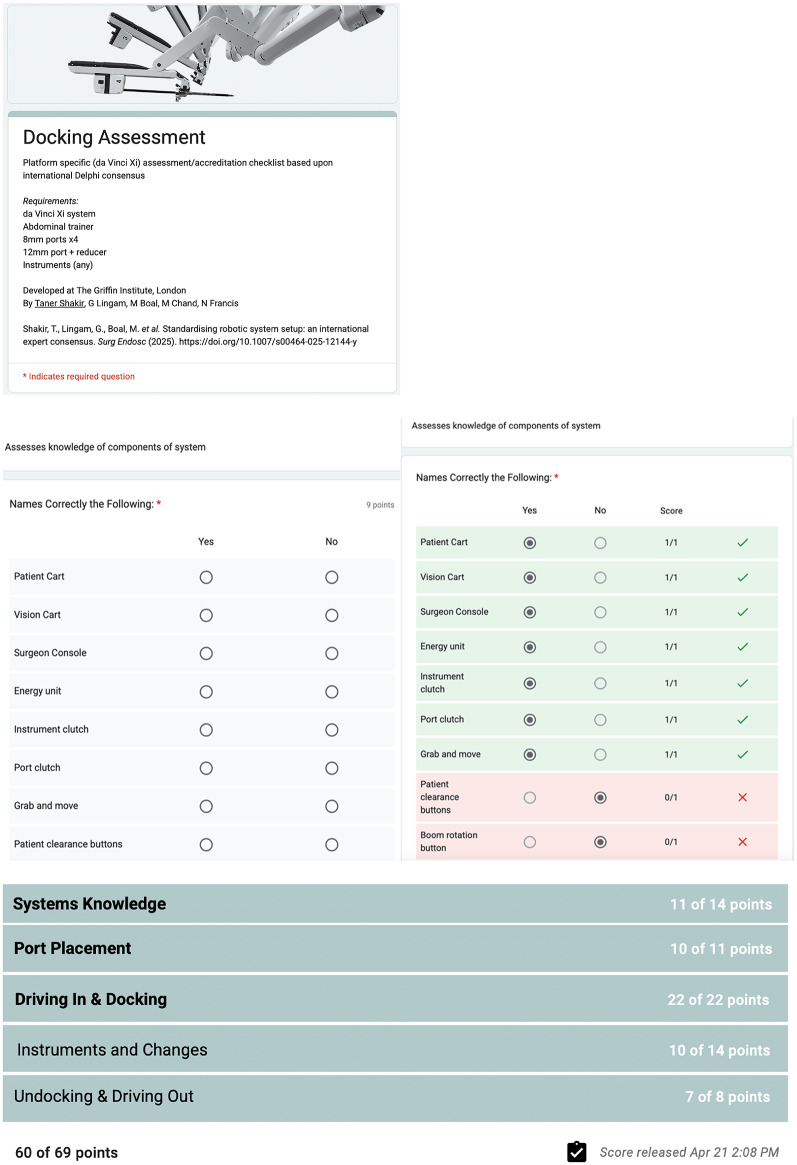
Online Docking Checklist Tool

### Assessment of docking and setup

Live assessments were conducted by a single ALSGBI-certified robotic trainer who had not been involved in the development of the checklist, the Delphi consensus, or the design of the present study. Participants were consented to video recording of their docking attempt with post event de-identification. This allowed for post-hoc review and also calculation of time taken for each docking attempt. Before the start of the study, the rater attended a standardised calibration session covering operation of the online form and the definitions for each checklist item, and undertook a calibration exercise in which two raters independently scored a pre-recorded simulated docking procedure to confirm alignment. Reliability of this was assessed with Cohen’s kappa and was calculated on per-item agreement between these two raters, however, given the single-video sample, a confidence interval was not derived Real-time scoring through the online form was used for each participant at both time points, eliminating manual transcription. The rater was aware of which assessment was the day 1 and which was the day 4 attempt.

### Inter-rater reliability

A post-hoc inter-rater reliability (IRR) analysis was performed using 12 videos prospectively recorded during the study, randomly selected from both the day 1 and day 4 time points. Two additional ALSGBI-certified robotic trainers, independent of the study team and of the development of the checklist, attended the same standardised calibration session as the live rater. Each independently scored the 12 videos using the 69-item checklist. Videos were anonymised with face blurring, and raters were blinded to time point and to the scores of the other raters. Total score agreement was quantified using the intraclass correlation coefficient (ICC; two-way random effects, absolute agreement, average measures) with 95% confidence intervals.

### GEARS dry-lab assessment

**Fig. 4:**
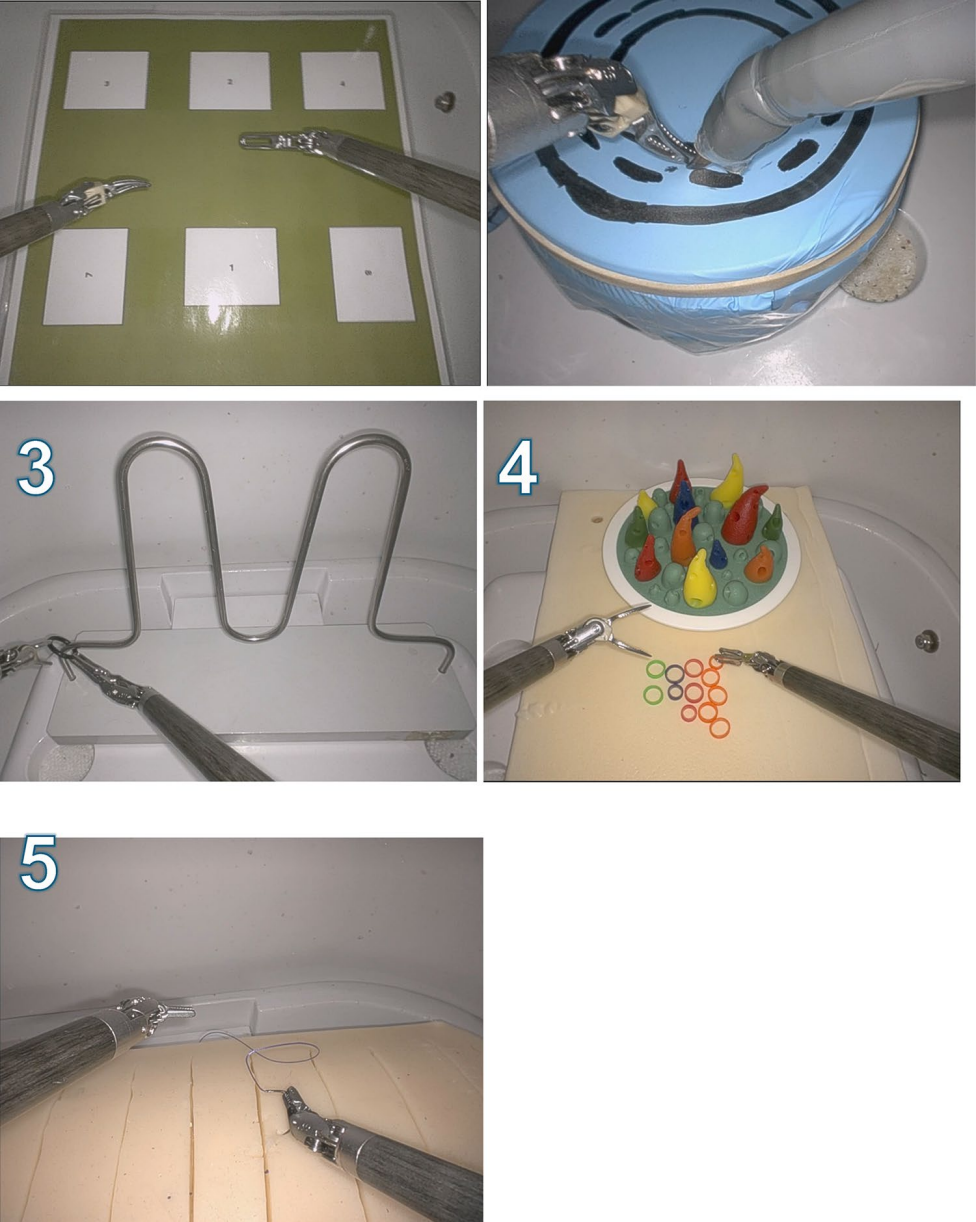
Dry Lab Skills Tasks. 1. Camera Target – sequential movement along a numbered board. 2. Glove Cut – Cut along the dotted line of a double layered glove, avoiding cutting glove beneath or out of plane. 3. Ring Rollercoaster – Move ring from right to left and then back from left to right without touching wire frame. 4. Sea Spikes – Pick and place task matching coloured rings onto coloured spikes. 5. Interrupted Suture – Single interrupted suture with surgeons knot

At both assessment time points (day 1 and day 4), participants performed five dry-lab tasks previously evaluated in our group’s robotic curriculum [9, 13]: camera targeting, sea spikes, ring rollercoaster, glove cut, and interrupted suture (Fig. 4). Each task was scored by trained course faculty using the Global Evaluative Assessment of Robotic Skills (GEARS) score [14, 15], a validated measure of robotic technical performance, with a maximum score of 30 per task; a composite score for each participant at both was calculated across tasks for both time points. Correlation with the docking checklist score was examined as an exploratory analysis, given that docking and basic console manipulation represent related but distinct tasks.

### Statistical analysis

Normality was assessed using the Shapiro-Wilk test. The majority of variables demonstrated non-normal distributions and non-parametric methods were therefore used throughout, with data presented as median and interquartile range (IQR). Change between time points was assessed using the Wilcoxon signed-rank test, with effect size expressed as the matched r statistic (r = Z/√N; small: 0.1, medium: 0.3, large: ≥0.5). Relative change was calculated as the change in median score compared with the day 1 median. Correlation between docking checklist scores and GEARS scores was examined using Spearman’s rank correlation coefficient (ρ). IRR was reported as the ICC with 95% confidence intervals. All analyses were performed using SPSS version 31 (IBM). Statistical significance was defined as *p* < 0.05.

## Results

### Demographics

Fifty-one surgical trainees and consultants participated (Table 1). The cohort had a mean age of 38.5 years (SD 7.7) and were predominantly male (64.7%). Participants ranged in experience levels: 24 consultants (47.1%), 19 senior residents (37.3%), and 8 junior residents (15.7%). Senior residents were defined as having at least four years of surgical experience. General surgery was the most common specialty (54.9%), followed by urology (23.5%). Participants had substantial backgrounds in minimally invasive surgery (mean 12.7 years of surgical training; mean 270.6 laparoscopic procedures performed independently), with no prior robotic experience.

**Table 1:** Participant Demographics

Variable	Total (*N* = 51)
**Age (years)**, Mean (SD)	38.5 (7.7)
**Gender**, n (%)	
Male	33 (64.7%)
Female	18 (35.3%)
**Hand Dominance**, n (%)	
Right	47 (92.2%)
Left	4 (7.8%)
**Nationality**, n (%)	
British	13 (25.5%)
International	38 (74.5%)
**Surgical Grade**, n (%)	
Consultant	24 (47.1%)
Senior Resident	19 (37.3%)
Junior Resident	8 (15.7%)
**Primary Specialty**, n (%)	
General Surgery*	28 (54.9%)
Urology	12 (23.5%)
Gynaecology	4 (7.8%)
Other**	7 (13.7%)
**Surgical Experience**, Mean (SD)	
Years of Surgical Training	12.7 (8.4)
Laparoscopic Procedures Assisted	295.6 (278.4)
Laparoscopic Procedures Performed	270.6 (252.1)

*Includes Colorectal, UGI, HPB/Liver Transplant, and Paediatric Surgery. **Includes Plastics,* Head and Neck*,* and Medical specialties*

### Inter-rater reliability

In the post-study IRR analysis using 12 procedure videos randomly sampled and scored independently by two additional blinded ALSGBI-certified raters, total-score agreement between the three raters were ICC = 0.91 (95% CI 0.75–0.97). The pre-study calibration exercise was calculated by two raters scoring the same simulated docking video had strong agreement (Cohen’s κ = 0.87). Given the single-video sample, a confidence interval was not derived.

### Change in score with training

Docking scores increased between attempts (Table 2). Median docking score improved from 43.0 (IQR 34.0–47.5; 62.3% of maximum) at the day 1 attempt to 58.0 (IQR 53.0–61.0; 84.1%) on day 4, a relative increase of approximately 35% (Wilcoxon signed-rank *p* < 0.001; *r* = 0.87, 95% CI 0.87–0.88). All five GEARS-scored dry-lab tasks similarly demonstrated significant improvement between attempts (composite GEARS median 18.60 [IQR 17.50–19.30] versus 25.80 [25.20–26.40], a relative increase of approximately 39%; *p* < 0.001; *r* = 0.87); individual task results are presented in Supplementary Table S2. Assessment time per attempt also decreased significantly, from a median of 25 min (IQR 24–26) on day 1 to 12 min (IQR 12–14) on day 4, a relative reduction of approximately 52% (*p* < 0.001; *r* = 0.87).

**Table 2:** Docking checklist score, composite GEARS score, and assessment time at both time points (*n* = 51). All p-values < 0.001 for between-attempt comparison by Wilcoxon signed-rank test; matched effect size *r* = 0.87 for all three measures

Variable	Day 1 median (IQR)	Day 4 median (IQR)
Docking score (max 69)	43.0 (34.0–47.5)	58.0 (53.0–61.0)
Docking score (%)	62.3% (49.3–68.8%)	84.1% (76.8–88.4%)
GEARS composite (max 30)	18.60 (17.50–19.30)	25.80 (25.20–26.40)
Time taken (minutes)	25.0 (24.0–26.0)	12.0 (12.0–14.0)

### Relations to other variables

Correlation between docking and composite GEARS scores was not significant at day 1 (Spearman ρ = −0.02, 95% CI − 0.29 to 0.26; *p* = 0.91). A weak but significant positive correlation emerged at day 4 (ρ = 0.31, 95% CI 0.04–0.54; *p* = 0.027). Of the individual dry-lab tasks, the interrupted suture task showed the strongest association with docking score at day 4 (ρ = 0.38, 95% CI 0.12–0.59; *p* = 0.006), followed by camera targeting (ρ = 0.29, 95% CI 0.02–0.52; *p* = 0.039). Tasks more reliant on object manipulation (ring rollercoaster, sea spikes, and glove cut) did not correlate significantly with docking performance (all *p* > 0.12). Full correlations are shown in Table 3.

**Table 3:** Spearman rank correlations between GEARS dry-lab task scores and docking scores. CI=Confidence Interval. *p < 0.05; **p < 0.01

Comparison	Spearman ρ (95% CI)	*p*-value
Composite GEARS (Day 1) vs. Docking (Day 1)	−0.02 (− 0.29 to 0.26)	0.91
Composite GEARS (Day 4) vs. Docking (Day 4)	0.31 (0.04 to 0.54)	0.027*
Interrupted suture (Day 4) vs. Docking (Day 4)	0.38 (0.12 to 0.59)	0.006**
Camera target (Day 4) vs. Docking (Day 4)	0.29 (0.02 to 0.52)	0.039*
Sea spikes (Day 4) vs. Docking (Day 4)	0.22 (− 0.06 to 0.47)	0.12
Ring rollercoaster (Day 4) vs. Docking (Day 4)	0.08 (− 0.20 to 0.35)	0.59
Glove cut (Day 4) vs. Docking (Day 4)	0.07 (− 0.21 to 0.34)	0.61

## Discussion

This study presents preliminary validity evidence for a Delphi consensus-derived checklist for robotic setup and docking on the da Vinci Xi platform, structured according Messick’s and Cook and Hatala’s framework and interpreted alongside the four validation inferences described by Kane [5, 7, 8]. The evidence is consistent with the use of the checklist in a structured training context, while important sources of evidence remain to be accumulated in subsequent work.

### Content

Content evidence rests on the prior international Delphi consensus, in which a multidisciplinary steering committee generated initial statements which were refined across rounds of voting by international expert respondents, and ultimately formed the 69 observable items used in the checklist. The diversity and experience of the panel, including consultant surgeons, surgical care practitioners with high case volume, multiple surgical specialties, and 13 international countries adds to the weight of the content. Furthermore, the rounds refining the statements provide a link between checklist content and docking competence. The relative number of items per phase additionally provides an implicit weighting, with port placement, driving in and docking, and instruments and changes having the highest number of checklist points. These phases represent some of the most critical aspects in setup, with the majority of errors reported to be occurring during the docking and instrument phases on a single centre workflow analysis [16].

### Response process

This evidence is supported by the binary scoring structure and the online form with real-time calculation. Furthermore, the granularity of the checklist reduces rater subjectivity, and has the ability to assess a wide range of performance. We nonetheless acknowledge that any human-rated assessment retains some element of subjectivity [17]; the checklist is therefore best characterised as a structured rather than purely objective assessment.

### Internal structure

The principal evidence comes from the post-hoc analysis whereby two additional independent, blinded ALSGBI-certified raters scored 12 procedure videos randomly sampled from the study cohort. The ICC was 0.91 (95% CI 0.75–0.97) for total scores across study performances. Moreover, the initial pre-study calibration exercise between two raters scoring the same simulated procedure yielded Cohen’s κ = 0.87 also provides evidence, albeit weaker, to this.

### Relations to other variables

Correlation with composite GEARS scores was absent at day 1 and modest (ρ = 0.31) at day 4. This pattern is consistent with docking and basic console skill being related but overall different. As participants progressed through the curriculum, performance on both improved, but the moderate correlation suggests that competence in one be a marker of competence in the other. Both remain relevant, complementary components of robotic competence, which the curriculum is designed to develop in parallel. With respect to the correlation, the interrupted suture task was the most strongly associated dry-lab task; the tasks emphasising pure object manipulation showed weak or absent correlation. These findings should be interpreted cautiously and are best regarded as exploratory. They support, rather than undermine, the case for a bespoke docking-specific assessment, as basic skills measures appear to be insufficient as a surrogate marker.

### Consequences

Docking scores improved with a large effect size (*r* = 0.87) and a relative increase of approximately 35% between the day 1 and day 4 attempts, indicating sensitivity to learning over the course of structured training. One must be careful, however, not to overinterpret this finding. The design does not isolate the effect of the checklist itself: all participants received the same training and were assessed with the same tool, and the live rater could not be blinded to the time point of each attempt. The change in scores indicates that the construct measured by the checklist responds to training, as would be expected of a useful learning-progress measure, but does not establish that use of the checklist drives or improves learning.

A median post-training day 1 score of 62.3% in a cohort with no prior robotic experience reflects, in part, the training participants would have just received, and the inclusion of accessible early items (such as correct identification of system components) appropriate to an entry-level assessment. A checklist that yielded near-zero scores at baseline would be of limited use for formative feedback and would not capture incremental learning across early items. The relatively narrow ceiling effect after a 4 day intensive robotic curriculum (median 84.1%) suggests scope for the tool to discriminate further at higher levels of skill, particularly when used with operators of varying experience.

Assessment time per attempt also decreased markedly between time points (median 25 to 12 min; approximately 52% reduction; *p* < 0.001; *r* = 0.87), moving congruently with the improvement in score. This provides convergent indirect evidence that the construct measured by the checklist responds to training, while the same caveats on inferring an educational effect of the checklist itself apply.

### Interpretation with Kane’s validation inferences

In addition to the applying Messick’s framework (Table 4), the evidence can also be applicable to Kane’s four inferences (scoring, generalisation, extrapolation, and implications) [8]. The scoring inference, that observations are converted accurately into a score, is supported by the binary checklist, operational definitions, and real-time online scoring that reduces transcription error. The generalisation inference, that scores generalise across raters and events, is supported principally by the IRR of independent blinded raters. The extrapolation inference, of which the test-setting performance reflects real-world practice, is partially supported by assessment on a functioning da Vinci Xi system, content derived from expert operative practice, and a modest convergence with basic skills tasks assessed with GEARS, a validated measure of robotic technical skill. The implications inference, that decisions based on scores are justified, remains the least developed: the evidence supports formative use, but does not yet justify accreditation.

**Table 4:** Summary of mapping of evidence to Messick’s five sources of validity evidence

Source of evidence	Evidence in this study	Current strength
**Content**	Delphi consensus of > 50 international experts from 13 countries	Established (prior work)
**Response process**	Binary observable items with online form reducing transcription error.	Supported
**Internal structure**	Inter-rater reliability of 12 videos with 2 blinded raters: ICC = 0.91 (95% CI 0.75–0.97).	Supported
**Relations to other variables**	Exploratory correlation with composite GEARS score after day 4 (ρ = 0.31, 95% CI 0.04–0.54)	Limited/indirect
**Consequences**	Score and time both responsive to training (*r* = 0.87)	Preliminary; further study required

### Limitations

Several limitations apply. The live rater could not be blinded to time point during participant assessment; the additional inter-rater work was undertaken specifically to provide internal structure evidence under blinded conditions, but this does not remove the inherent limitation of the live assessment design. The consequences analysis cannot demonstrate that the checklist itself produces an educational effect; it shows only that scores are sensitive to training. The convergent analysis used GEARS as a related but distinct comparator; assessment of relations to other variables would be strengthened by direct comparison across operators with differing docking experience. The study was also conducted at a single centre, and multi-centre replication is required to confirm generalisability.

Participants were medical practitioners only, limiting external validity to the surgical care practitioner population for whom the checklist may ultimately be most relevant. Furthermore, a 69 item checklist may appear too lengthy, however, items reflect discrete observable behaviours during a single attempt, and assessment time for second attempts averaged approximately 12–14 min per attempt with the online tool. Section-level scoring permits targeted formative feedback on individual procedural phases, consistent with the educational utility expected of a procedural checklist. The checklist is currently platform-specific (da Vinci Xi) and developed for abdominopelvic surgery; extension to other robotic systems and specialties is required. Stronger evidence for this source would come from comparison of checklist scores across operators with documented differences in docking experience, a study design we recommend for future work. Use of the checklist for accreditation would additionally require evidence not gathered here, including a defined pass/fail threshold derived through a formal standard-setting exercise, decision accuracy, test–retest reliability, and evidence that performance predicts safe clinical docking. Furthermore, no individual items are currently designated as safety-critical or “critical-fail”; and the designation of this would be a necessary step before use in accreditation.

## Conclusion

This study provides preliminary validity evidence, organised across the five sources of Messick’s framework and interpreted through Kane’s inferences, for a Delphi consensus-derived checklist for robotic setup and docking on the da Vinci Xi platform. Further evidence, particularly from multi-centres, additional raters and comparison across operator experience levels in the clinical setting, is required before the tool can be recommended for accreditation purposes. The present work provides a foundation on which such subsequent evidence can be built.

## Supplementary Information

Below is the link to the electronic supplementary material.

Supplementary Material 1

Supplementary Material 2

Supplementary Material 3

## Data Availability

No datasets were generated or analysed during the current study.
