# Supplementary material for: Preliminary validity evidence for a platform-specific assessment tool for robotic setup and docking
Source: J Robot Surg. 2026 Jul 20;20(1):669. doi: 10.1007/s11701-026-03589-x (PMC13385074; doi:10.1007/s11701-026-03589-x)
Supplement: Supplementary file 1 — Supplementary Material 1 [file 11701_2026_3589_MOESM1_ESM.pdf]

Docking Assessment

Platform specific (da Vinci Xi) assessment/accreditation checklist based upon international Delphi consensus

Requirements:
da Vinci Xi system
Abdominal trainer
8mm ports x4
12mm port + reducer
Instruments (any)

Developed at The Griffin Institute, London
By Taner Shakir, G Lingam, M Boal, M Chand, N Francis

Shakir, T., Lingam, G., Boal, M. *et al.* Standardising robotic system setup: an international expert consensus. *Surg Endosc* (2025). https://doi.org/10.1007/s00464-025-12144-y

* Indicates required question

1. Participant ID *

____

Systems Knowledge

Assesses knowledge of components of system

2. Names Correctly the Following: * 10 pc

Mark only one oval per row.

	Yes	No
**Patient Cart**		
**Vision Cart**		
**Surgeon Console**		
**Energy unit**		
**Instrument clutch**		
**Port clutch**		
**Grab and move**		
**Patient clearance buttons**		
**Boom rotation button**		
**Remote Centre**		

3. Ask to describe functionality of endoscope buttons and how to interact with endoscope * 4 pc

Mark only one oval per row.

	Yes	No
**Long press target button to target**		
**Short press target button to change eye video output**		
**Long press light button to toggle endoscope light**		
**30up and 30down orientation changes**		

Port Placement

Assesses understanding of principles of port placement

4. Ask to describe port placement and setup for a Pelvic procedure with robot approaching from (left/right) * 11 pc

Mark only one oval per row.

	Yes	No
**Identifies target anatomy based on operation (midpoint of surgical workspace)**		
**Knowledge endoscope port should be 10-20cm away from target**		
**Names and understands concept of remote centre**		
**Ports placed perpendicular to target**		
**Places ports 6-8cm apart (range 4 - 10cm)**		
**Assistant port minimum 7cm away from robotic ports**		
**Marks ports after establishing pneumoperitoneum**		
**Does not place ports less than 2cm from bony landmarks**		
**Does not place any da vinci/assistant ports between da vinci ports and target anatomy**		
**Considers performing laparoscopy prior to docking for setup**		
**Knowledge of reducer when using 8mm instrument in 12mm port**		

Driving In & Docking

Assesses ability to drive in and dock patient cart

Instruct candidate to proceed to drive robot in from patient cart touchpad

5. From Patient Cart *

9 pc

Mark only one oval per row.

	Yes	No
**Selects correct anatomy (upper abdomen, pelvic, thoracic etc)**		
**Selects correct cart location**		
**Presses and holds deploy for docking until completion**		
**Able to manually manipulate robot with joysticks**		
**Correctly grasps handlebars and drive enable switches**		
**Slowly drives to operating table monitoring patient clearance**		
**Ensures correct patient side as defined during deploy for docking**		
**Drives laser line within 5cm of initial endoscope port**		
**Uses reverse communication throughout with defined points of reference relative to patient position**		

6. Initial Docking *

7 pc

Mark only one oval per row.

	Yes	No
**Docks initial endoscope arm to initial endoscope port correctly**		
**Loops camera cable over endoscope to avoid hanging and tension**		
**Neutralises horizon**		
**Correctly long presses on target button at target anatomy**		
**Holds cannula to support during motion**		
**Ensures endoscope arm is parallel to blue vertical crosshair line on boom (or equivalent)**		
**Checks for obstructions when boom rotates during targeting**		

7. Subsequent Docking *

Mark only one oval per row.

	Yes	No
**Docks other arms correctly with no error message/sound**	◯	◯
**Docks medial arm first for ease (e.g 3 prior to 4)**	◯	◯
**Uses camera from a different port to check remote centre of initial endoscope port**	◯	◯
**Burps ports to release tension on abdominal wall**	◯	◯
**Ensures hand breadth between arms**	◯	◯
**Ensures correct spacing of arms at boom level**	◯	◯

Instruments and Changes

Assesses ability to safely insert and change instruments from patient side

Requires assessor to sit in console and request / manipulate instruments

8. Initial Instrument Insertion *

Instruct candidate to insert 1 or more instruments

Check working from console

Mark only one oval per row.

	Yes	No
**Confirms which instruments to be inserted into which arms with reverse communication**	⬭	⬭
**Inserts instrument under direct vision from trocar**	⬭	⬭
**Double checks remote centre location**	⬭	⬭
**Correctly gives control of all instruments to surgeon**	⬭	⬭
**Correctly gives control of camera to surgeon**	⬭	⬭
**Confirms diathermy connected if applicable and appropriately placed with reverse communication**	⬭	⬭
**Confirms robot at control of surgeon console with reverse communication**	⬭	⬭

7 pc

9. Instrument Changes *

Consider putting instrument into unsafe position

Instruct candidate to change "X" instrument in Arm "Y"

Mark only one oval per row.

	Yes	No
**Uses reverse communication for all applicable steps**		
**Confirms which instrument to be removed from which arm**		
**Confirms instrument not grasping anything**		
**Confirms instrument wrist straightened**		
**Inserts new instrument with guided tool change (green light)**		
**Confirms instrument inserted fully and control back to surgeon**		
**Understands instrument housing LED lights colours (eg flashing blue = control at patient cart)**		

Undocking & Driving Out

Instruct candidate to remove all instruments and undock

10. Undocking *

5 pc

Mark only one oval per row.

	Yes	No
**Confirms instruments and camera are removed prior to undocking port**		
**Removes trocars under vision to check for bleeding**		
**Turns off camera light to prevent fire risk**		
**Confirms patient clear prior to robot being driven away**		
**Instructs for robot to be driven away**		

11. Driving out *

3 pc

Mark only one oval per row.

	Yes	No
**Confirms safe to proceed with reverse communication**		
**Drives robot away correctly prior to sterile stow**		
**Sterile stows correctly**		
