# Supplementary material for: Preliminary validity evidence for a platform-specific assessment tool for robotic setup and docking
Source: J Robot Surg. 2026 Jul 20;20(1):669. doi: 10.1007/s11701-026-03589-x (PMC13385074; doi:10.1007/s11701-026-03589-x)
Supplement: Supplementary file 2 — Supplementary Material 2 [file 11701_2026_3589_MOESM2_ESM.docx]

**Supplementary Table S2**

GEARS individual task and composite scores at both attempts (n=51). Data as median (IQR). All p-values from Wilcoxon signed-rank test; r=matched effect size

| **GEARS Task** | **Attempt 1 median (IQR)** | **Attempt 2 median (IQR)** | **p-value** | **r** |
| --- | --- | --- | --- | --- |
| Camera Target | 18.0 (16.0–20.0) | 25.0 (24.0–26.0) | <0.001 | 0.870 |
| Sea Spikes | 20.0 (18.0–21.0) | 26.0 (24.0–27.0) | <0.001 | 0.870 |
| Ring Rollercoaster | 19.0 (15.0–20.0) | 26.0 (24.0–27.0) | <0.001 | 0.865 |
| Glove Cut | 18.0 (16.5–20.0) | 26.0 (25.0–27.0) | <0.001 | 0.870 |
| Interrupted Suture | 18.0 (17.0–19.0) | 28.0 (26.0–28.0) | <0.001 | 0.869 |
| **Composite (mean, max 30)** | **18.60 (17.50–19.30)** | **25.80 (25.20–26.40)** | **<0.001** | **0.870** |
